# Depression Is Associated with Cognitive Dysfunction in Older Adults with Heart Failure

**DOI:** 10.1155/2011/368324

**Published:** 2011-12-13

**Authors:** Sarah Garcia, Mary Beth Spitznagel, Ronald Cohen, Naftali Raz, Lawrence Sweet, Lisa Colbert, Richard Josephson, Joel Hughes, Jim Rosneck, John Gunstad

**Affiliations:** ^1^Department of Psychology, Kent State University, 221 Kent Hall Addition, Kent, OH 44242, USA; ^2^Department of Psychiatry, Summa Health System, Akron, OH 44304, USA; ^3^Department of Psychiatry and Human Behavior, Brown University, Providence, RI 02912, USA; ^4^Institute of Gerontology, Wayne State University, Detroit, MI 48202, USA; ^5^Department of Kinesiology, University of Wisconsin, Madison, WI 53706, USA; ^6^Harrington-McLaughlin Heart and Vascular Institute, University Hospitals Case Medical Center and Department of Medicine, Case Western Reserve University School of Medicine, Cleveland, OH 44106, USA

## Abstract

Persons with heart failure (HF) frequently exhibit cognitive impairment with deficits in attention and memory. Depression is common in HF though its possible contribution to cognitive impairment is unknown. Cognitive dysfunction and depression may share common mechanisms in HF, as both are associated with similar abnormalities on neuroimaging. A total of 116 participants with HF (68.53 ± 9.30 years) completed a neuropsychological battery and self-report measures of depression. Regression models showed depression incrementally and independently predicted test performance in all cognitive domains. Follow-up partial correlations revealed that greater depressive symptoms were associated with poorer performance on tests of attention, executive function, psychomotor speed, and language. These results indicate that depressive symptoms are associated with poorer cognitive performance in HF though further work is needed to clarify mechanisms for this association and possible cognitive benefits of treating depression in persons with HF.

## 1. Introduction

Heart failure (HF) is prevalent and now affects more than five million Americans [1]. Older adults with HF have high rates of mortality, hospital admissions, and debilitating symptoms such as shortness of breath, fatigue, and susceptibility to other medical problems [2]. Though less frequently examined, mental health issues are common in persons with HF. Many patients report decline in memory and other cognitive abilities and an estimated 25% to 75% of HF patients exhibit impairment on neuropsychological testing compared to normative data [3]. Deficits emerge in multiple cognitive abilities, specifically various types of memory [4, 5]. Executive functions such as attention and problem solving are also impaired. Similarly, depression is also common in persons with HF [6]. Though rates vary across studies, an estimated 21%–36% of heart failure patients can be diagnosed with depression, on the basis of both clinical diagnoses and elevated rates on various depression questionnaires [7, 8].

 Few studies have directly examined the association between cognitive impairment and depression in persons with HF despite the likelihood of common mechanisms. For example, persons with HF exhibit numerous pathological changes on neuroimaging, including greater atrophy and the presence of white matter hyperintensities, frequently in frontal brain regions [9]. In turn, these changes are associated with increased depressive symptoms in healthy older adults and other patient samples [10, 11]. Such findings suggest that depression is closely associated with cognitive impairment in persons with HF, and we examined this possibility in a sample of well-characterized older adults with HF. To further elucidate possible mechanisms for their co-occurrence, results from transcranial doppler (TCD) were also examined.

## 2. Methods

### 2.1. Participants

One hundred and sixteen adults diagnosed with HF were recruited from Summa Health System for this study (68.53 ± 9.30 years; 36.5% female). See Table 1. For inclusion, participants were between the ages of 50–85 years of age, English-speaking, and had an established diagnosis of New York Heart Association (NYHA) class II or III HF at the time of enrollment. Potential participants were excluded for history of significant neurological disorder (e.g., dementia and stroke), head injury with more than 10 minutes loss of consciousness, severe psychiatric disorder (e.g., schizophrenia and bipolar disorder), substance use, renal failure, and sleep apnea.

### 2.2. Instrumentation

#### 2.2.1. Neuropsychological Tests

Participants were administered a neuropsychological battery by trained research personnel under the supervision of a licensed clinical neuropsychologist to assess the following cognitive domains.

**Global Cognitive Function**

The modified minimental state examination (3MS) is brief screening measure that provides an estimate of global cognitive function through attention, memory, language, and spatial abilities [12]. A lower score on the test indicates poorer cognition.

**Attention and Executive Function**

Attention and executive function were assessed using the frontal assessment battery (FAB), the trail making test A (TMT-A), the trail making test B (TMT-B), and the symbol digit modalities test. The FAB is a short measure consisting of six subtests that assess aspects of executive function. Subtests include brief versions of similarities, lexical fluency, motor series task of attention, and tasks of motor inhibition. Higher scores reflect better performance [13]. Both the TMT-A and TMT-B ask participants to rapidly draw a connecting line between numbered circles (TMT-A) or alternating numbered and lettered circles (TMT-B) [12]. Longer completion time is indicative of worse cognitive performance. The symbol digit modalities test is a reliable and valid measure of visuomotor speed and complex attention [14]. The task requires speeded coding of numbers to correspond with symbols and lower scores illustrate worse performance.

**Memory**

The California verbal learning test-second edition (CVLT) and the complex figure test (CFT) were used to measure memory. The CVLT requires learning, recall, and recognition of a 16-item word list. This test incorporates indices of learning (sum of trials 1–5), immediate recall (short free recall), delayed recall (long free recall), and recognition (discrimination) [15]. For analyses these four scores were averaged into one overall CVLT score. The CFT requires copying, a short delay recall, and a long delay recall of a complex geometric design [16]. Again, both the short delay recall and long delay recall scores were averaged to create one overall CFT score. For both the CVLT and CFT higher scores indicate better performance.

**Language**

To measure language, the Boston naming test-II (BNT-II) and the Animal naming task were used. The BNT-II consists of 60 illustrations of objects ranging from high familiarity to low and asks individuals to name each object pictured [12]. Animal Naming asks individuals to generate as many animal names as possible within a given time limit [17]. Lower scores on both tests indicate poorer performance.

**Motor**

The grooved pegboard was used to measure complex coordination and speeded fine motor skill [12]. Individuals place notched pegs into a 5 × 5 board as quickly as possible. Time to completion for dominant and nondominant hand performance is recorded, and shorter duration represented better motor skills.

#### 2.2.2. Depressive Symptoms

The Beck depression inventory II (BDI-II) was used to measure the presence and severity of depression [18]. This measure is a 21-item self-report instrument and has good psychometric properties. Higher scores on the BDI-II indicate more severe depressive symptoms.

#### 2.2.3. Cardiovascular Fitness

To estimate current level of cardiac fitness, all participants completed the two minute step test (2MST) [19]. The test asks individuals to walk in place, lifting their knees to a target midway between their kneecap and the crest of the iliac. Participants were able to use a wall or chair for balance. A higher step count indicates better cardiovascular fitness.

#### 2.2.4. Cerebral Blood Flow

Transcranial doppler (TCD) was used to measure cerebral blood flow. After head measurements, participants completed a 10-minute rest period before an expanded STOP protocol was conducted [20]. Mean velocity and pulsatility was calculated for both the anterior cerebral arteries (ACA-V and ACA-P) and middle cerebral arteries (MCA-V and MCA-P).

### 2.3. Procedure

All procedures were approved by the local Institutional Review Board and participants provided written consent before enrollment. Participants first completed a 90-minute neuropsychological battery by trained research personnel under the supervision of a licensed clinical neuropsychologist in order to assess multiple cognitive functions as well as a 2-minute step test to evaluate cardiac fitness. A series of demographic, medical history, and self-report questionnaires, including the BDI-II, were then completed by all participants. Finally, participants completed the TCD evaluation within 2 weeks of this primary evaluation. 

### 2.4. Data Analysis

In order to compare across tests, raw scores were converted to T-scores using established normative data. Impairment for each test was operationalized as being >1.5 standard deviations below normative performance. Frontal, memory, and language composite scores were calculated by averaging the T-scores of neuropsychological tests on the basis of their respective composite category. Specifically, (1) the frontal composite score was comprised of the FAB, TMT-A, TMT-B, and the symbol digit modalities test, (2) the memory composite score was made up of the CVLT and CFT, and (3) the language composite score incorporated the BNT-II and the animal naming task. The motor functioning comprehensive score only used the pegboard task, whereas the global function category only comprised the 3MS.

 Separate linear regression models were preformed for each composite score as well as motor function and global cognitive functioning. Regression analyses were conducted in block format to determine whether depression was independently associated with cognitive test performance. The first block included demographic and medical control variables, specifically sex, hypertension, and cardiac fitness. BDI scores were then entered in the second block, and incremental prediction of the model to predict cognitive performance was examined. Finally, partial correlations adjusting for sex, hypertension, and cardiac fitness (2MST) were then conducted between depression and each individual neuropsychological test as well as TCD results to clarify regression findings. SPSS version 19 was utilized for all study analyses.

## 3. Results

### 3.1. Prevalence of Cognitive Impairment and Depressive Symptomatology

Patients demonstrated cognitive impairment on all neuropsychological tests. See Table 1. Approximately 19% of participants had an elevated score of 14 or more on the BDI-II, indicating clinically significant levels of depression [21]. 

### 3.2. Depression Is a Predictor of Cognitive Impairment in HF

Regressions were conducted between depression and the composite scores of each cognitive domain after adjusting for sex, hypertension, and cardiac fitness (62.39 ± 22.08 steps). Regression analyses showed BDI scores provided incremental prediction for all composite scores after adjusting for control variables, including global cognitive functioning (*F*(4,143) = 8.09, Δ*R*2 < .001, *P* < 0.001), attention/executive (*F*(4,143) = 12.41, Δ*R*2 = .03, *P* < 0.001), memory composite scores (*F*(4,143) = 3.04, Δ*R*2 = .03, *P* = 0.02), language composite scores (*F*(4,143) = 7.20, Δ*R*2 = .03, *P* < 0.001), and motor functioning (*F*(4,126) = 11.01, Δ*R*2 = .03, *P* < 0.001). See Table 2. 

### 3.3. Frontal, Language, and Motor Impairment in HF

To clarify these findings, partial correlations adjusting for sex, hypertension, and cardiac fitness between the BDI-II and specific neuropsychological test scores and TCD results were computed. Results showed multiple significant correlations between the BDI and cognitive test performance, including trail making test A (*r* = −.20, *P* = 0.04), trail making test B (*r* = −.32, *P* < 0.01), digit symbol coding (*r* = −.25, *P* = 0.01), grooved pegboard (*r* = −.21, *P* = 0.02), and the Boston naming test (*r* = −.25, *P* < 0.01). A trend was noted for animal naming (*r* = −.18, *P* = 0.05). There were no significant partial correlations between depression and 3MS, FAB, CVLT, and CFT or for the association between depression and TCD indices. See Table 3.

## 4. Discussion

Findings from the current study demonstrate that depression is independently associated with multiple tests of cognitive function in older adults with HF, including measures of attention, executive function, and language abilities. No such pattern emerged for tests of learning and memory. Several aspects of these findings warrant brief discussion.

 The prevalence of cognitive impairment within our sample is higher than that of healthy older adults and broadly consistent with past research in persons with HF [5]. Participants exhibited deficits on all cognitive tests, especially frontal and motor tasks. Cognitive deficits within this population often impact daily living and increase mortality [3, 22]. For example, recent work indicates that cognitive dysfunction is associated with reduced ability to complete activities of daily living in persons with HF [23]. Similarly, the current results also indicated a large number of participants to have clinically significant levels of depressive symptoms. As with cognitive impairment, depression confers many additional risks and is associated with poor outcomes in HF patients, including reduced compliance with medical regimen, higher risk for readmission, and greater mortality risk [24, 25]. Given the projected increase in HF prevalence, further work is much needed to develop improved treatments options and compensatory strategies for cognitive impairment and depression in persons with HF [26].

 Self-reported depressive symptoms on the BDI-II were independently associated with performance on all cognitive domains in this sample of older adults with HF not just tests of attention and executive function. Past work had shown that persons with major depressive disorder exhibit impairments on tests of attention, executive function, and visuospatial learning and older adults with depression show reductions on executive function and memory [27, 28]. The current results extend this work and suggest that depression is associated with reduced performance on global and specific measures of cognitive function in persons with HF.

 Several possible explanations for these findings exist, including the known impact of depression on speeded tasks such as the trail making test and motor performance [29]. However, a possible, alternative explanation is that depression exhibited by HF patients may be at least partly due to structural brain changes [24, 30]. Both depression and cognitive processes, such as speeded tasks, may be affected by structural brain changes resulting from cardiovascular disease, similar to other medical illnesses [11, 31]. For example, disruption in frontal-striatal pathways is associated with reduced function on tests of executive functioning and also with depressive symptoms in older adults [32–34]. Such findings may account for the poor success rates in traditional interventions for depression when used in persons with HF [35, 36], as it is possible that the depression in such samples is not an emotional reaction but rather a neuropsychiatric condition that emerges secondary to structural brain changes. Prospective studies that examine depression, cognitive function, and neuroimaging are much needed to clarify this possibility.

 An interesting finding from the current study is that while depression was associated with frontal/subcortical tasks, it was also strongly correlated with the BNT-II, a language task predominately mediated by temporal lobe functioning. Trends from another language-based task, animal naming, further support the association between depression and language functioning in persons with HF. One possible explanation for this pattern involves the possible damage to frontal-striatal pathways found in HF noted above, as past studies have linked reduced frontal functioning to poorer language function [37]. Given the age and medical characteristics of the current sample, this association may also reflect the contribution of early Alzheimer's disease (AD). Deficits in confrontation naming are often among the first deficits in persons with early stage AD, and the BNT-II is known to be sensitive to these deficits [38]. Additional work is much needed to better understand the possible association between depression and AD pathology in persons with HF.

 In brief summary, depression is correlated with cognitive impairment in HF patients, and future research is needed to clarify possible mechanisms, including the possible contribution of abnormalities on neuroimaging. A better understanding of the association between depression and cognitive impairment may improve treatment outcomes for HF patients. Medical professionals should be aware of the possible relationship between depression and cognitive function in HF, as the current findings suggest a high rate of co-occurrence. Care providers detecting one condition are encouraged to also screen for the other. Future studies are needed to clarify the possible effectiveness of screening for depression and cognitive function in persons with HF in reducing hospitalization and mortality rates. Such studies could reduce the high personal and financial burden of HF.

## Figures and Tables

**Table 1:** Demographic, medical, and cognitive characteristics of 116 older adults with HF.

Demographic	Mean (SD)	Percent
Age	68.53 (9.30)	
Female		36.5
Hypertension		70
Heart attack		55.4
Type 2 diabetes		34.1
CABG/bypass surgery		35.2
2-min step test	59.58 (22.90)	

Depression	Mean (SD)	Percent impaired

BDI	8.20 (7.66)	19

Cognitive function	Mean (SD)	Percent impaired

3MS	92.50 (5.72)	11
TMT-A	43.07 (18.70)	9.7
TMT-B	114.69 (56.43)	3.9
FAB	15.59 (2.56)	9.4
Digit Symbol	49.34 (14.93)	4.1
CVLT	38.67 (11.29)	4.1
CFT	25.35 (5.89)	7.1
BNT-II	53.63 (5.96)	8.3
Animal Naming	19.01 (4.90)	2.9
Pegboard	102.83 (25.33)	6.6

**Table 2:** Linear regressions between depression and cognitive composite scores.

Variable	B	SE	*β*	*t*	*P*	Δ*R^2^*
Frontal composite						
Control variables	.16	.03	.42	5.66	<0.001	.23
Full model	−.17	.08	−.17	−2.28	0.02	.03
Memory composite						
Control variables	.07	.03	.19	2.36	0.02	.07
Full model	−.12	.08	−.12	−1.50	0.14	.01
Language composite						
Control variables	.12	.03	.32	4.08	<0.001	.14
Full model	−.18	.08	−.17	−2.23	0.03	.03
Motor function						
Control variables	.20	.04	.43	5.38	<0.001	.23
Full model	−.25	.10	−.19	−2.43	0.02	.03
Global cognitive						
Control variables	.17	.03	.39	5.06	<0.001	.18
Full model	−.03	.10	−.02	−.26	0.78	<.001

Control variables included sex, hypertension, and cardiac fitness.

**Table 3:** Partial correlations among BDI, TCD results, and test performance.

Variable	*r*	*P*
Cognitive tests		
Global cognitive function		
3MS	.02	0.86
Attention and executive function		
TMT-A	−.20	0.04*
TMT-B	−.32	<0.01**
FAB	−.13	0.18
Digit symbol	−.25	0.01*
Memory		
CVLT	−.14	0.15
CFT	−.10	0.31
Language		
BNT-II	−.25	<0.01**
Animal naming	−.18	0.05
Motor		
Pegboard	−.21	0.02*
Transcranial Doppler		
MCA-pulsatility	−.01	0.88
MCA-mean velocity	−.11	0.25
ACA-pulsatility	.04	0.71
ACA-mean velocity	−.14	0.14

*P < 0.05. **P < 0.01. All correlations adjusted for sex, hypertension, and cardiac fitness.
